# Role of Cytokines and Chemokines in Vitiligo and Their Therapeutic Implications

**DOI:** 10.3390/jcm13164919

**Published:** 2024-08-20

**Authors:** Marcelina Kądziela, Magdalena Kutwin, Paulina Karp, Anna Woźniacka

**Affiliations:** Department of Dermatology and Venereology, Medical University of Lodz, pl. Hallera 1, 90-647 Lodz, Poland; marcelina.kadziela@stud.umed.lodz.pl (M.K.); kutwin1989@gmail.com (M.K.); paulina.karp@stud.umed.lodz.pl (P.K.)

**Keywords:** treatment, biologics, biological drugs, vitiligo, Janus kinase inhibitors (JAKs), cytokines

## Abstract

Vitiligo is a persistent autoimmune disease characterized by progressive depigmentation of the skin caused by the selective destruction of melanocytes. Although its etiopathogenesis remains unclear, multiple factors are involved in the development of this disease, from genetic and metabolic factors to cellular oxidative stress, melanocyte adhesion defects, and innate and adaptive immunity. This review presents a comprehensive summary of the existing knowledge on the role of different cellular mechanisms, including cytokines and chemokines interactions, in the pathogenesis of vitiligo. Although there is no definitive cure for vitiligo, notable progress has been made, and several treatments have shown favorable results. A thorough understanding of the basis of the disease uncovers promising drug targets for future research, providing clinical researchers with valuable insights for developing improved treatment options.

## 1. Introduction

**Figure 1:**
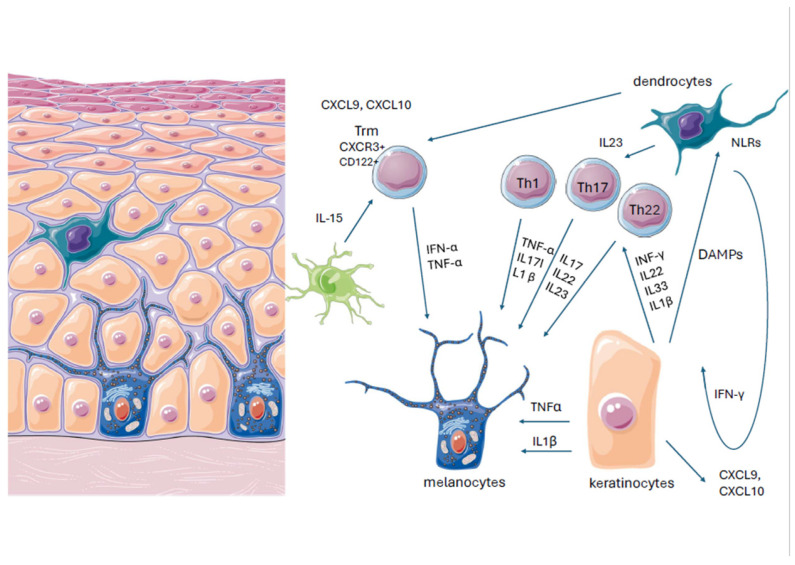
Role of main cytokines and chemokines in the pathogenesis of vitiligo.

Vitiligo is an autoimmune skin disorder diagnosed in 0.5–2% of the population [1]. The patient typically presents with chalky-white, well-demarked patches on the skin, which may occur in specific areas. As a consequence, vitiligo patients can also suffer reduced quality of life and serious psychological consequences [2]. Its pathogenesis is believed to be influenced by various genetic and metabolic factors, oxidative stress, melanocyte adhesion, and innate and adaptive immunity [3]. As a result of these different mechanisms, melanocytes are attacked by the immune system, and they lose intercellular connections, which leads to cell death. One notable aspect of vitiligo is the Koebner phenomenon (KP), a triggering factor where new lesions appear on areas of the skin that have been subjected to trauma or injury [4]. KP can be triggered by various factors, including physical trauma, chemical exposure, mechanical stress, and medical interventions such as laser therapies, which can provoke laser-induced KP reactions [5,6]. Also, diet and psychological stress have an influence on oxidative stress levels and may initiate the disease [7]. In vitiligo, the action of external and internal factors in individuals with genetic predispositions stimulates autoimmune processes in which CD8+ T cells exert a cytotoxic effect that causes the direct destruction of melanocytes [8]. While Treg cells suppress autoreactive CD8+ T cells in normal conditions, this regulatory function is impaired in vitiligo, and CD8+ T activation and proliferation remain unopposed [9]. The CD8+ T cells are most commonly directed against melan A, gp100, human tyrosinase-related protein-1 (TRP-1), TRP-2, and tyrosinase [10]. Autoreactive cytotoxic CD8+ cells promote melanocyte destruction and disease progression by local production of IFN-γ. Damaged melanocytes release DAMPs (damage-associated molecular patterns, which are identified by innate immune system cells, thanks to specific receptors: pattern recognition receptors (PRRs) and NOD-like receptors (NLRs)). Following activation, the NLRs create inflammasomes; these induce the discharge of caspase 1, IL-1β, and IL-18, which are involved in the death of melanocytes [11]. IL-1β activates Th17 cells and promotes Treg cell dysfunction. One DAMP, heat shock protein (HSP70), activates dendritic cells to produce IFN-α; these, in turn, stimulate keratinocytes to generate CXCL9 and CXCL10, which also damage melanocytes [12] (Figure 1).

Resident memory (Trm) cells represent a distinct subset of T cells whose role is to mediate long-term immune response in vitiligo. Trm cells have CD69, CD103, and CD49a on the surface, and when activated, they can secrete CXCR3, IFN-α, and TNF-α [13]. Trm cells expressing CD69 and CD103 markers are enriched in stable vitiligo skin in contrast to individuals experiencing active disease; this finding is consistent with their function as memory cells that endure even after the resolution of active inflammation [14].

While the pathogenesis of vitiligo is known to a certain extent, there is still no available effective method of treatment. Topical corticosteroids (TCS) and calcineurin inhibitors are used to obtain repigmentation, and a systemic corticosteroid is used to stabilize the active vitiligo [15]. TCS should be used with particular caution because they may cause contact allergy [16] and different adverse effects—atrophy of skin, telangiectasia, hypetrichosis, acneiform eruptions, cataract, and glaucoma—when they are applied on eyelids. Therefore, topical calcineurin inhibitors are recommended for lesions located on the face and for long-lasting therapy [17]. Most clinical trials test topical tacrolimus for vitiligo. Kumar and colleagues proved that tacrolimus stabilizes acral vitiligo and leads to the repigmentation of patches [18]. Pimecrolimus has a mild therapeutic effect on vitiligo and may be used as another option for treatment [19]. Nofal et al. compared the effectiveness of combined microneedling therapy and topical preparation. Combined microneedling and trichloroacetic acid (TCA) was more effective in the treatment of vitiligo than combined microneedling with either fluorouracil or pimecrolimus [20]. Psoralen plus ultraviolet-A radiation (PUVA) therapy has certain advantages, thanks to its immunosuppressive potential and ability to regenerate melanocytes, but its use is associated with phototoxic effects, nausea, and a higher risk of cancer [21]. A safer and more effective option than PUVA therapy is narrowband UVB (nb-UVB) phototherapy [22,23,24], and the combination of nb-UVB with topical treatment is currently the first-line option for clinicians [25,26,27]. However, this treatment does not result in complete repigmentation in all patients and is often a long-term option; furthermore, relapses are common within 12 months following discontinuation of therapy [28].

The aim of this manuscript is to review the contribution of different cytokines and chemokines in the development of vitiligo and examine the potential effectiveness of existing treatment options.

## 2. Materials and Methods

Articles were searched in the National Library of Medicine PubMed database. Inclusion criteria were studies reporting on vitiligo, written in English language with available abstracts. Case reports, reviews, original studies, case-control studies, cross-sectional studies, and randomized controlled trials were taken into account. The molecules we considered were IL-6, IL-15, TNF-α, IL-1β, IL-22, IL-17, IL-23, IFN-γ, and their therapeutic implications. All articles were collected and grouped into subsections.

## 3. Results

The review covered the importance of the above-mentioned molecules in the pathogenesis of vitiligo and drugs that have targets in these molecules and are currently available or are tested in clinical trials.

### 3.1. IL-6

IL-6 is a versatile cytokine with pro-inflammatory effects produced by lymphocytes and macrophages. Together with IL-8, it recruits immune cells to the skin and directs them to target melanocytes [29], and with the inflammatory cytokines TNFα and IL-1β, it has been found to inhibit melanocyte proliferation [30]. IL-6 is believed to act through two molecules: the IL-6 receptor IL-6R and gp130. When IL-6 binds to mIL6R, the membrane-bound form of IL-6R, it forms a complex comprising IL-6, IL-6R, and gp130; this complex activates Janus kinases (JAKs), thus triggering two main signaling pathways involved in autoimmune diseases [31]. Indeed, patients with vitiligo have been found to demonstrate higher IL-6 serum concentrations than controls [32,33]. Moreover, as the level of IL-6 in sera positively correlates with disease progression, it can be regarded as a sensitive marker of active vitiligo [34,35].

#### IL-6 Inhibitor

IL-6 may inhibit melanogenesis [36] and thus halt disease progression and initiate melanocyte repigmentation. One such treatment involves the use of tocilizumab, a humanized monoclonal antibody targeting the IL-6 receptor. Contraindications to this drug include severe, chronic, and/or recurrent infections, tuberculosis, cancers, and hypersensitivity to tocilizumab [37]. Although only one case report of the positive effect of tocilizumab on vitiligo could be found in the literature, the results indicated resolution of facial and periungual vitiligo in a patient treated for seronegative rheumatoid arthritis [38]. Tocilizumab therapy has been found to increase serum IL-6 levels following IL-6R blockage; this increase could potentially have systemic effects, such as exacerbating vitiligo by intensifying the imbalance between Tregs and Th17 cells [39]. Indeed, paradoxical cases of halo naevi developing during tocilizumab therapy have been reported [36]. While there is some low level case-based evidence to suggest some benefit, the drug is not recommended for such indication [40].

### 3.2. IL-15

IL-15 belongs to the family of cytokines characterized by a four α-helix bundle structure. It shares the receptor components CD132, i.e., the cytokine receptor γ-chain, and CD122, i.e., the β-chain (IL-2/IL-15Rβ) with IL-2, another key cytokine [41]. In vitiligo, oxidative stress activates NF-κB signaling, thus inducing the expression and trans-presentation of IL-15 in keratinocytes; this enhances the activation and expression of cytotoxic proteins in CD8+ cells by activating both the STAT3 and STAT5 pathways. IL-15 maintains the signals of Trm cells. These can endure for an extended period and be reactivated with the same antigen, promoting disease relapses [42]. IL-15 expression was elevated in both the perilesional skins and blood circulation of individuals with vitiligo, indicating increased levels of locally-expressed and secreted IL-15 in these patients [43]. IL-15 is believed to play a crucial role in the survival of T-cells in the skin of individuals with vitiligo.

#### IL-15 Inhibitors

Ex vivo studies indicate that IL-15 supports Trm function and that IL-15-deficient mice exhibit impaired Trm formation. Trm cells express the CD122 subunit of the IL-15 receptor, and keratinocytes enhance the expression of CD215, which is essential for presenting the cytokine on their surface to activate T cells. Blocking IL-15 signaling with an anti-CD122 antibody effectively halted disease progression in mice with established vitiligo. Short-term administration of anti-CD122 inhibited Trm production of IFN-γ, while long-term treatment led to the depletion of Trm cells from skin lesions [44].

Targeting IL-15 or its receptor could serve as a prospective approach to developing tailored treatments that inhibit interactions between oxidative stress and the emition of IL-15 by keratinocytes. AMG 714 binds to IL-15, preventing it from binding with the IL-15 receptor. The use of this drug in vitiligo is being investigated in an ongoing phase IIa clinical trial (NCT04338581) [8].

### 3.3. TNF-α

Another important pro-inflammatory cytokine involved in inflammation, cell proliferation, differentiation, and apoptosis is TNF-α [45]. TNF-α is directly involved in melanocyte apoptosis, inhibition of melanogenesis, and melanocyte stem cell differentiation. It also increases the cytotoxic reaction toward melanocytes [46]. IFN-γ and TNF-α stimulate keratinocytes to produce matrix metallo-proteinase 9 (MMP-9), involved in destabilizing detachment between melanocytes by releasing the soluble form of E-cadherin [47]. In many studies, vitiligo patients demonstrated higher mean serum TNF-α levels than controls, with the level being correlated with disease activity [33,35,48]. These observations were confirmed by De et al., who found patients to have elevated TNF-α levels in serum and in lesional skin compared to controls [32].

#### TNF-α Inhibitors

TNF-α blockers such as adalimumab, infliximab, and etanercept have been used in various autoimmune and inflammatory conditions [49]. Contraindications to this drug group are sepsis, abscess, tuberculosis, optic neuritis, anaphylaxis, cancers, and congestive heart failure [50]. It is possible they may also be of value against vitiligo as TNF-α blockage may modulate the immune response and potentially inhibit the progression of vitiligo spots. While TNF-α inhibitors can stabilize progressive vitiligo in some patients [51], they may also be associated with new-onset vitiligo in others. Most of these cases were observed after the use of adalimumab and infliximab [52,53,54]; however, it remains unclear whether these are comorbidities or possible side effects. A 2007 pilot study found etanercept to be ineffective at treating vitiligo in all four test cases [55], although researchers reported a mild improvement in vitiligo lesions during treatment [56]. In addition, a cohort study involving 11,442 patients found anti-TNF agents to significantly increase the risk of vitiligo but not alopecia areata [57].

### 3.4. IL-1β

The formation of IL-1β interleukin depends on the activation of two of the NLRs that are part of the inflammasome: NLRP1 and NLRP3 [58]. Being a pro-inflammatory cytokine, IL-1β can trigger rapid release of iNOS and prompt a substantial amount of NO in tissues [59]. Increased IL-1β levels were observed in the sera of active-vitiligo patients, suggesting that IL-1β may play a role in dysregulating melanocytic activity in lesional skin [60]. It has been reported that NLRP1 and IL-1β immunostaining in perilesional skin was significantly associated with the dynamicity of the disease, and surprisingly, it added significantly more information on the disease progression with regard to the simple lymphocytic infiltrates [61].

### 3.5. IL-22

IL-22 is a member of the IL-10 family and plays a crucial role in a range of inflammatory and infectious diseases. Th22 and Th17 cells are major sources of IL22 [62]. IL-22 influences IL-1β production by activating NLRP3-caspase-1; it also plays a minor role in melanocyte proliferation and melanogenesis and enhances the synthesis of antimicrobial peptides and chemokines in human keratinocyte cells (HaCaT) [63]. The IL-22–IL-22R system predominantly activates STAT3 via the JAK-STAT pathway. It has been found that in patients’ sera with localized vitiligo, IL-2 levels were significantly elevated, while IL-17 and IL-22 levels were notably higher in patients’ sera with generalized vitiligo.; however, no correlation was found between IL-22 levels and disease activity or severity [35].

#### IL-22 Inhibitor

Antibodies neutralizing IL-22 have yielded promising results against psoriasis and rheumatoid arthritis, and they may improve the response to chemotherapy, especially in reducing metastases [64]. No studies have evaluated the effectivity of IL-22 antibodies in vitiligo.

### 3.6. IL-17

Research has indicated that Th17 cells might play a role in the development of vitiligo [65]. IL-6, TGF-β, and IL-23 drive the specialization of Th cells into Th17 cells, which then produce various specific cytokines, including IL-17A, IL-17F, IL-22 [66], and TNF-α [67]. In vitiligo patients, increased concentrations of serum IL-17 have been detected [60]. Some papers have also demonstrated significant positive correlations between IL-17 levels in serum and lesional skin and the activity, extent, and severity of vitiligo [65,68,69]. Furthermore, IL-17 reveals CCL20, a chemokine that can introduce cytotoxic CD8+ T cells into peripheral tissues from the systemic circulation; these cells are responsible for melanocyte destruction in vitiligo [70]. In addition, in inflammatory vitiligo, IL-17 stimulates keratinocytes to release chemokines that cause an influx of neutrophils, macrophages, and dendritic cells, which can also play a role in melanocyte destruction [71].

Th17 cells can be transformed into Th17.1 cells, which demonstrate a shared phenotype of Th17 and Th1 cells, or into Th17ex cells, i.e., transformed Th17 cells that produce IFN-γ instead of IL-17 [72]. These may constitute a novel potential therapeutic target for vitiligo treatment, as recent studies confirm that serum Th17.1 cell levels are elevated in vitiligo patients [73] and that these numbers fall after starting effective treatment [70].

#### IL-17A Inhibitors

Secukinumab is a monoclonal antibody that specifically blocks the action of pro-inflammatory interleukin-17A (IL-17A) and thus modulates the immune response. Studies have found it to be effective in managing various immune-mediated inflammatory disorders, such as psoriasis, psoriatic arthritis, and ankylosing spondylitis [74]. In one study, a 63-year-old man who developed vitiligo while undergoing treatment with adalimumab experienced resolution of both vitiligo and psoriasis after changing treatment to secukinumab [75]. A case report from 2021 also indicated that a patient developed new-onset vitiligo after administration of secukinumab therapy for psoriatic arthritis, but the vitiligo spots slowly began to repigment after one year of therapy [76].

However, some reviews and case series of new-onset vitiligo in individuals undergoing treatment with secukinumab report negative effects [77,78,79]. A pilot study evaluating the effectiveness of secukinumab in treating active non-segmental vitiligo did not find that IL-17 or Th17 cells had an exact role in the pathogenesis of vitiligo; however, it indicated that the balance of Th17/Th17.1/Th1 can change according to disease activity. One promising treatment option for vitiligo may, therefore, involve modulating the differentiation of Th17 toward Th17/1 and Th1 [73].

Another IL-17A blocker is ixekizumab, which is also widely used in various autoimmune conditions. A case study of a man suffering from psoriasis and psoriatic arthritis found that treatment with adalimumab caused whitening of the hair on the scalp and face, which was reversed after switching to ixekinumab [80]. Interestingly, though, most other such studies have reported new cases of vitiligo occurring during ixekizumab therapy [81,82,83]. Absolute contraindications to IL-17 inhibitors comprise allergic reaction to the drug, active IBD, and planned live vaccination [84].

### 3.7. IL-23

IL-23 is a heterodimer composed of the p19 and p40 subunits; it contributes to the expansion, evolution, and differentiation of Th17 lymphocytes [85]. The IL-23 receptor, consisting of IL-12Rβ1 and IL-23R, is expressed by inflammatory macrophages and DCs. The binding of IL-23 to the receptor leads to antigen presentation by DCs, formation of Th17 cells, and production of IFN-γ. IL-23 is believed to have a central role in autoimmunity, and that its dysregulation or overactivation may contribute to the progression of vitiligo [86]. IL-23 serum levels have demonstrated a positive association with the duration, activity, and extent of the non-segmental generalized vitiligo [67].

#### 3.7.1. IL-23 Inhibitor

Tildrakizumab, an IL-23 blocker, is effective and widely used in the treatment of psoriasis, rheumatoid arthritis, and inflammatory bowel diseases (IBD) [87]. Patients with vitiligo commonly demonstrate increased levels of IL-23 [85,88], and as such, it may represent a therapeutic target in vitiligo. Only one study examined the use of tildrakizumab in rapidly-progressive vitiligo: its use resulted in a 55% reduction in the Vitiligo Area Scoring Index (VASI) over 12 months [89].

#### 3.7.2. IL-12 and IL-23 Inhibitor

Ustekinumab is a monoclonal antibody that targets the p40 subunit common to IL-12 and IL-23, thereby preventing these cytokines from binding to the IL-12Rβ1 part of their receptors [90]. The drug targets Th1 and Th17 cells, which alter immune cell hypersensitivity and could hence offer potential benefits in dermatological conditions such as vitiligo, alopecia areata, and cutaneous systemic lupus erythematosus [91]. Although IL-23 induces the production of Th17 cells and mediates autoimmunity in vitiligo by influencing the secretion of the IL-17 family [67], little is known about the use of IL-12/23 blockers in practice. Elkady et al. report an impressive improvement in comorbid alopecia areata and vitiligo in a patient with psoriasis [92]. A nationwide retrospective multi-center study investigated the appearance of new-onset vitiligo during biologic therapy in patients treated for different reasons. Three patients developed new onset vitiligo under ustekinumab treatment, and one patient observed no effect in their existing vitiligo. Patients with new onset vitiligo were described as showing improvement during ustekinumab treatment. Surprisingly, one repigmentation was reported after switching from adalimumab to ustekinumab [93]. Another study on the drugs and therapeutic subclasses associated with vitiligo reported an association between vitiligo and ustekinumab therapy [94]. However, it is important to note that while some studies have shown ustekinumab to obtain positive responses in terms of repigmentation, the overall data and consensus regarding its effectiveness require further investigations.

IL-23 inhibitors should not be used in those with an allergic reaction to the drug and in active infections but are safe for patients with comorbidities such as diabetes mellites, obesity, or cardiovascular diseases [95].

### 3.8. IFN-γ

IFN-γ is mainly produced by plasmacytoid dendritic cells (pDCs). This cytokine interacts with Janus Kinase 1 (JAK1) and JAK2 on keratinocytes, thus activating the signal transducer and activator of transcription protein (STAT) [9]. Then phosphorylation occurs, and STAT moves to the nucleus to initiate the transcription of particular genes [96]. The JAK-STAT pathway has a role in the expression of CXCL9/10/11, i.e., chemokine ligands 9/10/11, that direct T-cells to the epidermis [97,98]. Moreover, these molecules mediate the recruitment of CD8+ T cells via chemokine receptor 3 (CXCR3); these are responsible for the apoptosis of melanocytes [35,47,99]. The results of a comparative study indicate that CXCL10 and IFN mRNA expression was notably elevated in non-lesional and perilesional skin in patients with vitiligo compared to healthy controls. Interestingly, the mRNA expression levels of CXCL10, and particularly IFN-γ, were elevated in the non-lesional skin of patients with active disease when compared to those with stable disease, which may suggest that these molecules are related to disease activity [100]. However, recent studies have found that in contrast to tissue, patients with vitiligo show decreased IFN-γ serum levels in comparison to controls [32,101].

#### 3.8.1. JAK Inhibitors

Currently, JAK inhibitors offer great promise for the treatment of vitiligo. The JAKs, *viz.* JAK1, JAK2, JAK3, and TYK2 are a family of cytoplasmic tyrosine kinases (TYKs). JAK1 and JAK2 modulate the transduction signal after IFN-γ binds to its receptor [102], and as such, the downstream IFN-γ/CXCL10 signaling pathway may be a potential therapeutic target in vitiligo. Recent studies indicate that JAK1 and JAK3, but not JAK2, demonstrated higher cutaneous expression in vitiligo skin compared to healthy skin [103,104]. Inhibiting the JAK/STAT pathway interferes with the detachment of low E-cadherin melanocytes in the basal layer of the epidermis. Additionally, it reduces the secretion of MMP-9 by keratinocytes in response to IFN-γ and TNF-α. Elevated levels of MMP-9, found in the skin and sera of vitiligo patients, contribute to the disruption of E-cadherin in the basal layer of the epidermis. Inhibition of MMP-9 prevents melanocyte detachment in vitro and in vivo [105]. Contraindications for oral JAK inhibitors include allergy to another JAK inhibitor, pregnancy, severe liver and kidney disease, blood disorders, cancers, active infections, active tuberculosis, and immunosuppressive therapies [106]. The main JAK inhibitors that are used in the treatment of vitiligo are described below.

##### Ruxolitinib

This molecule is an inhibitor of JAK1 and JAK2. The US Food and Drug Administration (FDA) approved topical ruxolitinib for repigmentation of non-segmental vitiligo in July 2022 for people aged 12 years and older. The effectiveness and safety of this drug was evaluated in phase III clinical trials: TRuE-V1 and TRuE-V2. The study involved patients 12 years of age or older with non-segmental vitiligo and depigmentation covering up to 10% or less of body surface area (BSA). Treatment with 1.5% ruxolitinib cream gave greater repigmentation than vehicle control over 52 weeks, but it was associated with acne skin lesions and pruritus [107]. The drug is believed to act by blocking the JAK1/2 pathway, which interferes with STAT transcription; the pathway itself mediates various inflammatory cytokines, growth factors, interferons, and interleukins [108]; however, the exact mechanism of the action remains unclear. One study comparing the effects of ruxolitinib cream and vehicle and examining the correlation between the level of CXCL10, an immunity biomarker, in skin and VASI scores, is currently underway [109]. It has been found that reducing the CXCL10 level may limit melanocyte destruction by reducing the inflow of CD8+ T cells [110]. Oral ruxolitinib is used by the FDA and EMA for treating myelofibrosis, polycythemia vera, and acute graft-versus-host disease [111]. Several studies indicate that while these drugs have a good effect on repigmentation in vitiligo patients, the effect disappears after discontinuation of treatment [112].

##### Tofacitinib

Tofacitinib, an inhibitor of JAK1/3, has been approved by the US FDA for psoriatic arthritis and shows promise for treating plaque psoriasis [113]. It was found to successfully inhibit CXCL10 secretion through the suppression of JAK-STAT signaling in keratinocytes [114]. The first use of oral tofacitinib in vitiligo was reported in 2015 in a case of generalized vitiligo involving approximately 10% of the body surface. Treatment resulted in significant repigmentation: vitiligo patch cover fell to only 5% after five months of treatment [115].

Oral and topical treatments have both yielded positive results, especially in areas of great sun exposure [114,116]. In two patients suffering from vitiligo with facial involvement, a combination of tofacitinib and low-dose, narrowband UVB radiation resulted in significant repigmentation [77]. These findings indicate that tofacitinib may require additional nb-UVB phototherapy to be more effective and to stimulate melanocytes to seed the epidermis, where tofacitinib can suppress the immune response [117]. So far, no clinical trials have investigated the efficiency and safety of tofacitinib in vitiligo patients, and further research is needed in this area.

##### Baricitinib

Baricitinib is a small molecule that mainly acts on JAK1/2. It is currently approved for rheumatoid arthritis but can be used in other conditions, such as atopic dermatitis or systemic lupus erythematosus [118]. One preliminary study on four patients with progressing vitiligo found that a combination of baricitinib and high-dose ultraviolet B irradiation caused significant repigmentation without any serious side effects [119]. The effectiveness and tolerance of oral baricitinib with phototherapy are currently being compared to phototherapy in an ongoing randomized clinical trial phase II (NCT04822584), and the results appear promising [47].

##### Ritlecitinib

Ritlecitinib is an orally-administered biological drug that inhibits JAK3 and the TEC kinase family [120], which may block cytokine activity and decrease the cytotoxic activity of CD8+ T cells. A randomized phase 2b clinical trial found the treatment to be effective against active non-segmental vitiligo over 48 weeks with good tolerance [121]. Phase III clinical trials of ritlecitinib in different age groups with active and stable vitiligo are ongoing (NCT05583526, NCT06072183, NCT06163326).

##### Ifidancitinib

Ifidancitinib is a JAK1/3 inhibitor that is currently under investigation in phase II clinical trials for the topical treatment of vitiligo [122]. This drug is able to induce depletion of effector function of T cells and has been used for treating alopecia areata [123]. The effectiveness of 0.46% ifidancitinib solution for treating non-segmental facial vitiligo is under examination (NCT03468855) [99].

##### Brepocitinib

Brepocitinib is an oral JAK1 and TYK inhibitor that is used in moderate and severe plaque psoriasis. A phase 2b study (NCT03715829) has explored the use of both ritlecitinib and brepocitinib for treating vitiligo, with and without phototherapy [99]. However, there are currently no trials exploring this drug in vitiligo.

##### Upadacitinib

Upadacitinib is an oral selective JAK1 inhibitor. Recent studies indicate a notable improvement in VASI and DLQI scores in patients treated with upadacitinib [124]. Its adverse events and effectiveness are being evaluated in a phase III clinical trial (NCT06118411).

##### Cerdulatinib

Cerdulatinib demonstrates specificity toward JAK1/3 and spleen tyrosine kinase (SYK) [125]. It has been demonstrated to be effective against B-cell malignancies, including diffuse large B-cell lymphoma, Burkitt lymphoma, and chronic lymphocytic leukemia (CLL) [126]. Cerdulatinib (0.37% gel) has been assessed for safety, tolerability, and systemic exposure in adults with vitiligo in the two-phase clinical trial (NCT04103060); however, the results are not available.

##### Delgocitinib

Delgocitinib is a JAK1/2/3 and TYK2 inhibitor. It has been found to be effective against vitiligo vulgaris after topical application [127].

Results of drugs used in the treatment of vitiligo are summarized in Table 1 and Table 2.

## 4. Other Perspectives

Vitiligo development is influenced by the IFN-γ-CXCL9/10-CXCR3 pathway, and new therapeutic strategies are being designed to take advantage of this. A 2005 study found IFN-γ neutralizing antibodies to induce repigmentation in two of four vitiligo patients who received intradermal perilesional injections [128]. It was also found to reverse vitiligo in mice [129]. CXCL10 is raised both in the skin and serum of vitiligo patients, and CXCR3, its receptor, is expressed on pathogenic T cells. In addition, minimal depigmentation is noted in patients with T cells lacking CXCR3, as well as in mice deficient in CXCL10 or treated with a CXCL10-neutralizing antibody. Neutralizing CXCL10 led to repigmentation in mice with established, widespread depigmentation [99]. Vitiligo patients also demonstrate increased CXCR3 levels in T cells in the blood and present CXCR3+ cells in skin biopsies. It was found that CXCR3-depleting antibodies can reverse vitiligo by reducing autoreactive T cell numbers [130].

Latanoprost is a prostaglandin F2alpha analog primarily used to reduce intraocular pressure in patients with open-angle glaucoma and ocular hypertension. As it induces hypertrichosis and hyperpigmentation due to increased melanogenesis, it may be suitable for treating hypopigmentation disorders such as vitiligo [131]. It can be used in monotherapy or in combination with other therapies. The combination of latanoprost with nb-UVB therapy is currently under evaluation for treating non-segmental vitiligo in a phase 4 clinical trial (NCT04811326).

Afamelanotide is the first synthesized analog of α-melanocyte-stimulating hormone (α-MSH). It appears more stable and active than the natural hormone [132]. Its efficacy and safety for inducing repigmentation are under evaluation in patients with vitiligo in two clinical trials: the first as monotherapy (NCT05210582) and the other in combination with nb-UVB light compared to nb-UVB light alone (NCT06109649).

Piperine, an alkaloid found in black pepper (Piper nigrum), has been studied for its potential therapeutic effects in treating vitiligo by promoting the proliferation of melanocytes [133]. It can be used in monotherapy or in combination with other therapies such as nb-UVB phototherapy, 308-nm excimer laser, and prostaglandin F2α analog [133,134,135]. Nb-UVB treatment combined with a topical piperine restored blood vessels in nearly all macules, leading to improvements in the Vitiligo Noticeability Scale (VNS) and repigmentation of white patches [135]. Piperine may offer a less aggressive alternative for vitiligo treatment compared to current options.

Another promising therapy is the intralesional administration of methotrexate every two weeks. This method appears to be effective among patients with focal vitiligo based on a pilot study; however, further studies on larger groups are necessary [136].

## 5. Conclusions

As cytokines and chemokines play such important roles in the development of vitiligo, their mechanism of action should also contribute to its treatment. Biologic and small molecule therapies are effective in various immune-mediated inflammatory diseases, including vitiligo; however, they can also provoke vitiligo onset or exacerbate pre-existing disease. Many existing treatments are nonspecific. While our growing understanding of the underlying processes of vitiligo has paved the way for more targeted approaches, the data regarding their mechanisms of action and their efficacy remain broad. JAK inhibitors offer particular promise as treatments, and this is supported in ongoing clinical trials. Nevertheless, there continues to be a need for further research into new and more effective treatments.

## Figures and Tables

**Table 1:** Targets and effects of drugs used in the treatment of vitiligo.

Drug	Target	Effect
Tocilizumab [36,37,38,39,40]	IL-6 receptor	Lack of effectiveness, new vitiligo lesions (case reports)
anti-CD122 [44]	IL-15	Repigmentation of vitiligo lesions (mice model)
IL-15 monoclonal antibody (AMG 714) [8]	IL-15	Ongoing phase IIa clinical trial
Adalimumab Infliximab Etanercept [49,50,51,52,53,54,55,56,57]	TNF-alpha	Increased risk of new-onset vitiligo, controversial therapeutic results (case reports, cohort study)
-	IL-1β	-
IL-22 neutralizing antibody [64]	IL-22	-
Secukinumab [75,76,77,78,79]	IL-17A	Controversial results (case reports)
Ixekizumab [80,81,82,83,84]	IL-17A	New vitiligo lesions (case reports)
Tildrakizumab [89]	IL-23	Insufficient studies (case report)
Ustekinumab [91,92,93,94]	IL-12 and IL-23	New vitiligo lesions, controversial therapeutic results (case reports, case/non-case study/review)
Ruxolitinib [107,108,109,110,112]	JAK1/2	Good clinical response, repigmentation of vitiligo lesions (approved by FDA and EMA in adults and adolescents from 12 years of age with non-segmental vitiligo)
Tofacitinib [115,116,117]	JAK1/2/3	Repigmentation of vitiligo lesions, nbUVB may increase clinical effect (case reports, retrospective case series)
Baricitinib [119]	JAK1/2	Repigmentation of vitiligo lesions, nbUVB may increase clinical effect (phase II clinical trial)
Ritlecitinib [121]	JAK3/TEC	Repigmentation of vitiligo (phase III clinical trial)
Ifidancitinib [99]	JAK1/3	Repigmentation of vitiligo lesions (phase II clinical trial)
Brepocytinib [99]	JAK1/TYK	No results (phase II clinical trial)
Upadacitinib [124]	JAK1	Repigmentation of vitiligo lesions (phase III clinical trial)
Cerdulatynib	JAK1/3	No results (phase II clinical trial)
Delgocitinib [127]	JAK1/2/3, TYK2	Good clinical response (case reports)

**Table 2:** Results of the most significant clinical trials for the treatment of vitiligo.

Drug	Study Name	Phase	Population	Primary Outcomes	Key Findings
Ruxolitinib	NCT04530344 (TRuE-V1)	Phase 3	Adolescents and adults with vitiligo	Percentage change in F-VASI (face) at Week 24	Significant improvement in facial vitiligo area scores; FDA approved ruxolitinib cream for vitiligo
Ruxolitinib	NCT04530357 (TRuE-V2)	Phase 3	Adolescents and adults with vitiligo	Percentage change in F-VASI (face) at Week 24	Confirmed efficacy and safety; supports use of ruxolitinib cream in broader population
Baricitinib	NCT04822584	Phase 2	Adults With progressive vitiligo	No result posted	No result posted
Ritlecitinib	NCT03715829	Phase 2b	Adolescents and adults with vitiligo	F-VASI improvement at week 24	Showed efficacy in repigmentation; generally well tolerated.
Ifidancitinib	NCT03468855	Phase 2	Adults with non-segmental facial vitiligo	F-VASI improvement	Change in the facial Vitiligo Area Scoring Index (F-VASI) score from baseline to Week 24.
Brepocitinib	NCT03715829	Phase 2	Adolescents and adults with vitiligo	No result posted	No result posted
Upadacitinib	NCT04927975	Phase 2	Adult Participants with Non-Segmental Vitiligo	No result posted	No result posted
Upadacitinib	NCT06118411	Phase 3	Adult and Adolescent Participants with Vitiligo	Is ongoing	Is ongoing
Cerdulatinib	NCT04103060	Phase 2	Adults with Vitiligo	No results posted	No results posted
